# The neonatal continuous glucose monitoring paradox: why at-risk infants still lack access to a transformative technology—A call for needs-led innovation

**DOI:** 10.3389/fmed.2026.1904160

**Published:** 2026-08-07

**Authors:** Alexandra Duncan, Hannah Burgess, Kathryn Beardsall

**Affiliations:** 1 Clinical School, University of Cambridge, Cambridge Biomedical Campus, Cambridge, United Kingdom; 2 Neonatal Unit, University of Cambridge Addenbrooke's Hospital NHS Trust, Cambridge, United Kingdom; 3 Department of Paediatrics, University of Cambridge, Cambridge Biomedical Campus, Cambridge, United Kingdom

**Keywords:** continuous glucose monitoring, equality of access, innovation, neonate, technology

## Abstract

Glucose instability in the newborn is common, often clinically silent, and carries serious consequences for neurodevelopment. With rising rates of prematurity and maternal diabetes, the number of infants at risk is growing. Two decades of research have established continuous glucose monitoring (CGM) as feasible in neonatal populations, with the potential for detecting previously missed dysglycaemic episodes alongside reducing the need for repeated invasive blood sampling. Despite this no CGM system is licensed for use in children below 2 years of age. Research in neonates has relied on off-label use of devices designed for adults, with challenges of insertion and regulatory barriers to use in clinical practice. Even the current off-license workaround is under threat as the commercial CGM design is moving toward integrated devices that combine sensors and transmitters. This development will make CGM more difficult to use in newborns, and unsafe in extremely preterm infants. Not only is this an example of inequity of access to those with significant clinical need it is also an innovation opportunity. This lack of needs-led innovation is not unique to CGM but is a case study of a broader issue affecting high-risk, and rare-disease populations. In these settings commercial incentives alone usually fall short and support for derisking development is essential. The EU and FDA have begun building regulatory infrastructure to address this. However, ensuring equitable access to innovations in care, of which CGM is just one example, will require sustained collaboration between clinical teams, research funders, regulators, and industry.

## Highlights

- No CGM device is licensed for use in newborn infants, despite two decades of evidence demonstrating feasibility, clinical utility, and potential cost-effectiveness in neonatal populations.
- This absence reflects the challenges of licensing preventing the generation of clinical data, which removes commercial incentive to develop neonatal-specific devices.
- The EU and FDA have begun to address this through dedicated rare diseases and paediatrics expert panel; the UK currently lacks a formal orphan or paediatric device designation or adapted evidence standard.
- Co-ordinated action from clinicians, NIHR, regulatory agencies, and industry is required to ensure equality of access to healthcare technology.

## Introduction

In the last 20 years continuous glucose monitoring (CGM) has dramatically changed the lives of many individuals living with diabetes mellitus who were at risk from both hyperglycaemia and hypoglycaemia. More than improved glucose control it has reduced the frequency of painful blood glucose testing and the fear of hypoglycaemia (1, 2). However newborn infants who are also at risk from dysglycaemia are still dependent on repeated heel prick blood sampling. This method is painful, leads to cumulative blood loss, and captures only a handful of point measurements in a population where glucose can be very labile (3). NICE now recommends continuous glucose monitoring (CGM) for adults and children with diabetes (4). However, no CGM system is designed or licensed for use under the age of 2 years. The need for glucose monitoring in the newborn encompasses the range from term infants at risk of hypoglycaemia, to extremely preterm infants at risk from both hypoglycaemia but more typically hyperglycaemia. Both have been associated with worse long-term outcomes (5, 6).

Newborns most at risk of hypoglycaemia are infants born to mothers with diabetes mellitus or having had treatment with beta blockers, as well as babies born preterm or small for gestational age (7, 8). Recurrent and severe hypoglycaemia is associated with impaired executive function and visual processing in childhood even when clinically silent (5, 9). Hyperglycaemia is often seen in babies with hypoxic ischaemic encephalopathy (HIE), but is most common in extremely preterm infants and is also associated worse health outcomes (10–12).

Two decades of research have highlighted that CGM can detect dysglycaemic episodes in the newborn that standard monitoring misses and can support clinical management (Table 1). However, all this research has been undertaken with CGM systems that have been designed and developed for adults and that are challenging to use in the newborn. This lack of a system designed and licensed for neonatal use impairs clinical research and the potential for CGM to support clinical care. Even this off-label workaround is currently under threat as the commercial CGM design is shifting toward integrated composite devices that cannot safely be applied to preterm infants. In contrast CGM is available to healthy adults to monitor the effect of lifestyle choices on glucose metrics, ahead of robust evidence for impacts on health outcomes (13, 14).

**Table 1:** Key neonatal CGM studies: summary of design, population, and findings.

Study/year	Design	Sample size	Population	Device	Blinded/real-time CGM	Primary endpoint	Key finding	Key limitations
**Very-low-birth-weight neonates**								
Beardsall et al. (25)	Feasibility, single-center	*n* = 16	VLBW (< 1500 g) preterm requiring intensive care within 24 h of delivery	Medtronic MiniMed	Blinded		CGM feasible in NICU; well tolerated; continuous profiles generated; no safety signals	Relatively low number of infants; insufficient paired values at hypoglycaemic level to assess agreement
Mola-Schenzle et al. (32)	Prospective cohort	*n* = 41	VLBW (< 1500 g) preterm infants on full enteral nutrition	Medtronic Guardian	Real-time		Glucose instability persisted despite clinical stability and fully established enteral feeds	Relatively low number of infants, though association between parenteral nutrition duration and glucose variability remained independent after adjustment
Uettwiller et al. (22)	RCT, two arms	*n* = 48 (CGM *n* = 25; control *n* = 23)	VLBW (< 1500 g) preterm requiring intensive care within 24 h of delivery	CGM group - Medtronic MiniMed Guardian RT; Control group -Medtronic Guardian Clinical	Both. CGM group used real-time. Control group used blinded CGMS analyzed retrospectively	To compare the number of hypoglycaemic episodes per patient detected by intermittent capillary blood glucose testing vs. by CGM	Intermittent testing missed significantly more hypoglycaemic episodes than CGM (1.2 vs. 0.4/patient); CGM group had shorter hypoglycaemia duration; clinically silent hypoglycaemia revealed by blind CGM in standard care group	Imbalance in hypoglycaemia risk factors between groups (maternal-fetal infection only present in the CGM group)
Galderisi et al. (55)	RCT, two arms	*n* = 50	≤ 32 wks or ≤ 1500 g, < 48 h after birth	Dexcom G4 Platinum	Both. CGM group used real-time. Control group used blinded CGMS analyzed retrospectively	Percentage of time spent in the eugluycaemic range (72–144 mg/dL)	MARD within acceptable range; adapted insertion technique required for neonatal use	Lack of power to detect effect of CGM-guided glucose titration on clinical outcomes (blinded group had higher, non-significant number of adverse clinical outcomes)
Thomson et al. (56)	Accuracy and single-center pilot RCT of efficacy	*n* = 40 (Accuracy *n* = 20; CGM *n* = 10; control *n* = 10)	Very preterm < 1200 g, < 48 h after birth	CGM group -Enlite sensor, Medtronic Paradigm VEO; Control group—Medtronic iPro 2	Both. CGM group used real-time. Control group used blinded CGMS analyzed retrospectively	Percentage time in target range (2.6–10 mmol/L)	CGM with paper guideline increased time in target range vs. standard care; proof-of-concept for REACT	Possible bias between study groups (iPro 2 masked CGM uses different algorithm to Paradigm VEO); Relatively low number of infants
Beardsall et al. (31)	Feasibility RCT, two arms	*n* = 20 (Closed-loop *n* = 10; Control *n* = 10)	Very preterm < 1200 g, < 48 h after birth	Closed-loop system: Enlite sensor linked to Medtronic Paradigm VEO	Real-time used in both arms. Study group used closed loop, control group used paper algorithm	Percentage time in target range (4–8 mmol/L) between study arms	Closed-loop trebled time in target range vs. CGM alone; first automated glucose control in neonates	Small sample size led to some baseline differences between study groups despite randomization
REACT: Beardsall et al. (27)	Multicentre RCT (13 NICUs), two arms	*n* = 182 (CGM *n* = 84; control *n* = 95)	≤ 1200 g, ≤ 33+6 wks, < 24 h after birth	Medtronic Enlite sensor with Guardian 2 transmitter	Both. CGM group used real-time. Control group used blinded CGMS analyzed retrospectively	Percentage time in target range (2.6–10 mmol/L) between study arms in the first week of life	CGM reduced prolonged hyperglycaemia and hypoglycaemia; increased time in target glucose range; staff and parents reported CGM improved clinical care	Potential recruitment bias from short recruitment window; Lack of power to show clinical effect of difference in hyperglycaemia
Petrou et al. (28)	Health economic analysis		REACT population				CGM cost-dominant: lower costs and better outcomes; high probability of cost-effectiveness	
Galderisi et al. (33)	Observational	*n* = 22, (496 procedures)	Very preterm ≤ 32 wks and/or ≤ 1500 g, < 48 h after birth	Medtronic Enlite		Coefficient of glucose variation before and after the procedure	CGM revealed glucose variability as real-time marker of physiological stress during NICU procedures (nappy changes, heel sticks, venepunctures)	Secondary analysis of a small cohort
**Neonates at-risk of hypoglycaemia**								
Harris et al. (22)	Observational	*n* = 102	At-risk neonates, >32 wks	Medtronic MiniMed	Blinded		CGM detected far more hypoglycaemic episodes than intermittent testing; glucose variability characterized	Unclear of clinical impact of increased detection of hypoglycaemia
Wackerna-gel et al. (34)	Accuracy	*n* = 20	At-risk neonates, >34+ 6 wks, >2500 g	Medtronic Paradigm VEO	Blinded	CGM compared to POC	CGM accurate and feasible; provided earlier detection of hypoglycaemia and reduced procedural pain; calibration delay in neonates identified as important caveat for clinical use	Relatively low number of infants
Galderisi et al. (3)	Cochrane review of CGM use in preterm	Four RCTs enrolling 300 infants	Preterm neonates of any birth weight and any postnatal age admitted to NICU	Multiple	Mixed	Long-term neurodevelopmental outcomes	CGM provides valuable physiological insights; all studies off-label; no licensed neonatal device	None of the trials reported on neurodevelopmental outcomes
Myat Win et al. (54)	Retrospective cohort	*n* = 14 (9 with congenital hyperinsulinism)	Term and preterm persistent hypoglycaemia; (>48 h)	Dexcom G4/Medtronic Enlite and Paradigm	Real-time		CGM revealed glucose fluctuations in CHI undetectable by intermittent sampling	Data collection was dependent on retrospective review of clinical notes
**Neonates with neonatal encephalopathy**								
Tam et al. (57)	Prospective cohort + diffusion tensor imaging	*n* = 102	Term neonates with NE/HIE admitted to NICU	72 h of monitoring: Enlite connected to Medtronic iPro2	Blinded		Higher maximum glucose on day 1 associated with widespread brain microstructural changes on DTI	Use of therapeutic hypothermia may have limited detected of hypoglycaemia-associated changes
Kamino et al. (19)	Prospective cohort	*n* = 108	Term neonates with NE/HIE admitted to NICU	72 h of monitoring: Enlite connected to Medtronic iPro2	Blinded		Intermittent testing missed 50% of dysglycaemic events; CGM >10.1 mmol/L in 48 h linked to brain injury and adverse 18-month outcomes	Use of therapeutic hypothermia may have limited detected of hypoglycaemia-associated changes
Parokaran Varghese et al. (58)	Prospective cohort	*n* = 96	Term neonates with NE/HIE admitted to NICU	Enlite with Medtronic iPro2 + EEG for 48 h	Blinded		Hyperglycaemia associated with worse EEG brain function; CGM+EEG enables real-time study of dysglycaemia and brain function	Recording of concurrent EEG and CGM started >6 h after birth, potentially missing some early glucose variability

This state of play we term the “CGM paradox”: the imbalance between clinical need and availability of the medical technology. This paradox is not unique to CGM but is an example of the wider challenges faced for children with rare diseases or who are critically unwell in whom medtech development can be viewed as commercially unattractive or unviable. The challenge is often balancing the potential financial rewards against the barriers to device development and regulation. Here we describe the clinical need, the evidence of potential utility, the challenges of using off-license CGM devices as well as the requirements for neonatal design and development. We frame this not just to highlight the challenges but to identify areas of potential opportunity, and the importance of collaborative working to benefit patients and to support a needs-led approach to innovations in care.

## Glucose dysregulation in the neonatal period

Following birth, all newborns must adapt rapidly from continuous controlled maternal glucose supply to independent metabolic regulation. Even in healthy newborns this can result in a transient physiological fall in blood glucose. However, for a significant number of infants this period of hypoglycaemia can be prolonged and associated with relative hyperinsulinism, placing them at risk of neurological impairment. Newborns most at risk of hypoglycaemia are infants born to mothers with diabetes mellitus or having had treatment with beta blockers, as well as babies born preterm or small for gestational age (7, 8). These risk factors are increasing in prevalence in the population, and up to one in three at-risk neonates may develop hypoglycaemia during this transitional period (8). Hypoglycaemia is frequently clinically silent, making recognition dependent on active monitoring as follow-up studies have shown that even silent hypoglycaemia is associated with an increase in impaired executive function and visual processing at 4.5 years of age (5), and reduced literacy in middle childhood (9). The need for better monitoring was highlighted in a report from the Eunice Kennedy Shriver National Institute of Child Health and Human Development on “Knowledge gaps and research needs for understanding and treating neonatal hypoglycemia” (15).

Hyperglycaemia is common in extremely preterm infants with a daily prevalence of up to 30% (>10 mmol/l/180 mg/dl) in the first 2 postnatal weeks. Although prevalence then declines it can persist in some babies until time of discharge (16, 17). In these infants hyperglycaemia is associated with necrotising enterocolitis, retinopathy of prematurity, bronchopulmonary dysplasia, and intraventricular hemorrhage (10, 12). Population-level data from the Swedish EXPRESS cohort demonstrate that hyperglycaemia was associated with a more than twofold increase in adjusted 28-day mortality (OR 2.55; *p* = 0.005), and that insulin treatment was associated with lower mortality (17). Insulin treatment in these babies however remains controversial as variable insulin sensitivity increases the risk of hypoglycaemia. In this context CGM has the potential to provide early warning of falling glucose concentrations and reduce the need for such frequent heel prick blood samples (18). Infants with HIE characteristically can experience a combination of hyperglycaemia and hypoglycaemia, both of which independently predict death and adverse neurodevelopmental outcome (11). Glucose fluctuations are an early marker of neonatal stress and potential sepsis, and glucose is monitoring is a core part of neonatal intensive care.

However, monitoring is limited to either sampling from central arterial lines (possible in the first week) or repeated heel prick sampling which is painful. Both add to a cumulative blood loss in small fragile infants. More significantly the data provides only infrequent point measurements separated by hours, leaving clinicians blind to the glucose trajectories that occur between measurements. In a prospective study of 108 term neonates with neonatal encephalopathy undergoing both CGM and standard blood sampling, intermittent testing missed 50% of all hypo- and hyperglycaemic events on the first day of life (19). Clinical decisions are being made with only limited data and outcome studies are being conducted without fully capturing the glycaemic exposures that may matter most. The true burden of neonatal dysglycaemia is almost certainly greater than current data suggest, but the benefits and risks of interventions to optimize glucose control cannot be fully realized without a better way of monitoring glucose in this population (20).

## Evidence from CGM in neonates

The potential value of CGM in monitoring neonates has led to increasing literature exploring its accuracy, usability and efficacy in populations ranging from term babies at risk of hypoglycaemia to extremely preterm infants in NICU. These studies span two decades and include a range of devices used both masked and to guide clinical care (Table 1) (21).

For many years, the Harding group in New Zealand has used masked CGM in infants at risk from hypoglycaemia to provide an unbiased measure of exposure, both in relation to intervention and to neurodevelopmental outcomes. Their cohort studies have used CGM in approximately 400 term babies, with no reported adverse device effects, but revealed a link between clinically silent hypoglycaemia with worse neurodevelopmental outcomes in childhood (5, 22**?** ).

The feasibility of using CGM in babies undergoing therapeutic hypothermia following HIE has also been explored. These studies highlighted the wide variation in glucose control and risk of late hypoglycaemia (23), as well as the challenges of accuracy at low glucose thresholds in babies where peripheral circulation may be compromised (24). Among 108 term neonates with neonatal encephalopathy, CGM-detected hyperglycaemia was associated with specific patterns of MRI brain injury and adverse neurodevelopmental outcomes at 18 months (19).

Early feasibility studies confirmed that CGM sensors were well tolerated in preterm infants in intensive care, with continuous glucose profiles generated without safety signals (25). This led to an international trial in preterm infants where masked CGM highlighted both hypoglycaemic and hyperglycaemic to be much more prevalent than clinically recorded (26). Following this, REACT, an international randomized controlled trial, demonstrated that real-time CGM reduced exposure to prolonged hyperglycaemia and hypoglycaemia, with infants in the CGM group spending longer in the target glucose range (27). Health economic analysis of the REACT data demonstrated that CGM was cost-dominant: lower costs and better outcomes, and robust across sensitivity analyses (28). Other single center trials have shown real-time CGM can reduce the number of hours exposed to hypoglycaemia alongside reducing the number of painful heel pricks by 25% (18).

Groups using CGM in very low birth weight babies have shown the relationship between gestational age and glycaemic variability (29), and used CGM data to create percentile curves for “normative” glucose measurements in very low birth weight babies in NICU (30). A pilot of closed-loop glucose control with machine learning also demonstrated that automated CGM-guided insulin delivery increased time in target range three-fold compared with CGM alone (31). CGM has also been important in demonstrating that even clinically stable preterm babies are at risk from recurrent glucose fluctuations despite being fully established on enteral nutrition (16, 32).

**Figure 1:**
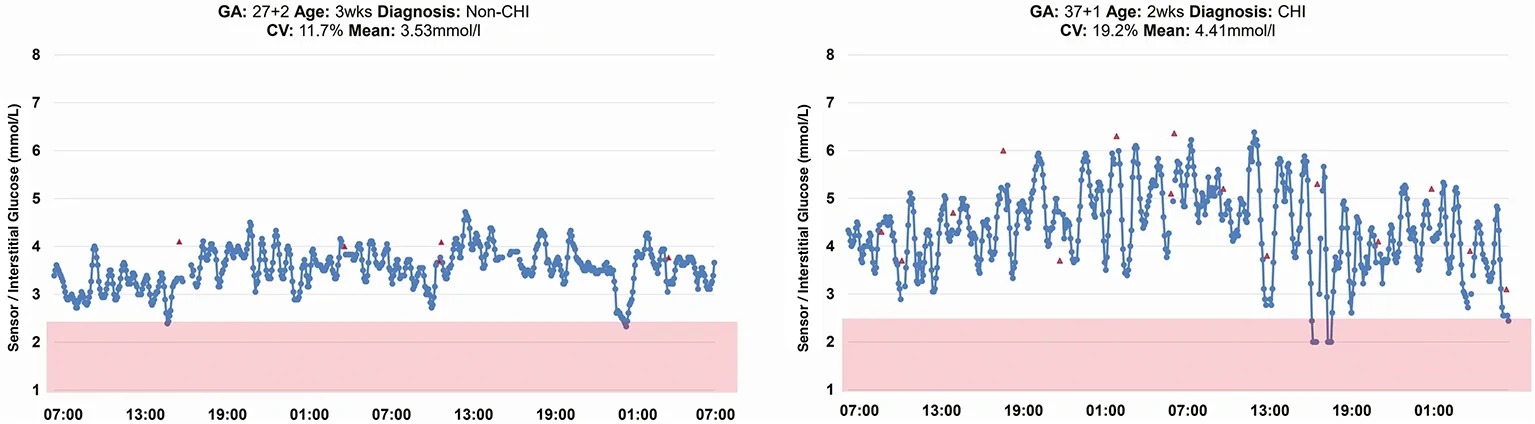
Example of continuous glucose monitoring traces. Comparison of continuous glucose monitoring patterns of a preterm and term baby with hyperinsulinism using Medtronic CGM in real time mode. Both babies were on full hourly enteral feeds and the term baby was on diazoxide. Patterns are suggestive of more rapid fluctuations in glucose levels in the term infants with congenital hyperinsulinism compared with the preterm infant. GA gestational age. CHI congenital hyperinsulinism CV coefficient of variation. Adapted from Win et al. (54).

The wealth of data from CGM has provided physiological insights: with glucose variability rising measurably during routine NICU procedures including heel prick and venipuncture (33), revealing information about physiological stress responses, potential early sepsis signals, and individual metabolic patterns that intermittent sampling simply cannot capture. There are significant differences in glucose profiles between preterm babies, term babies and those with different clinical conditions (Figure 1). Accuracy has improved steadily across device generations (34), with a recent study reporting a mean absolute relative difference (MARD) of approximately 7% in preterm infants (35), although there are concerns about accuracy at extremes where clinical decisions are more important. Systematic reviews suggest that CGM provides valuable physiological insights and signals clinical benefit across neonatal populations, while noting the limitations of small sample sizes and the absence of a licensed neonatal-specific device (3, 36).

## Neonatal specific device requirements

Neonatal CGM use currently carries practical challenges distinct from adult application and clinical use. There are a range of CGM systems all with different sensors, modes of insertion, reported accuracy and connectivity, but none address the needs of the newborn. Fragile skin particularly in preterm infants increases the risk of adhesive-related injury, very limited subcutaneous tissue means sensors cannot be inserted at 90° and the size of limbs means standard inserters cannot be held stable for insertion in the smallest at risk babies. The limited point accuracy and lag in measurement in comparison to blood glucose concentrations means CGM are useful for providing trends over time, particularly where glucose is labile, but need interpretation together with confirmatory blood glucose testing. The current glucose ranges on CGMs do not tend to read below 2.8 mmol/l which is challenging where the threshold for intervention for neonatal hypoglycaemia in some units is 2.6 mmol/l and others as low as 2.0 mmol/l. Accuracy in the low glucose range is key limitation. Alarm fatigue is a further risk in an environment already saturated with monitoring alarms, so safe use depends on adequate staff training and clear guidelines. In addition, the long-term clinical benefits of interventions based on the data available from CGMs remains to be evaluated (20). However, appropriately powered clinical trials are practically impossible without a device better designed for the newborn, and even smaller studies are becoming more challenging.

**Figure 2:**
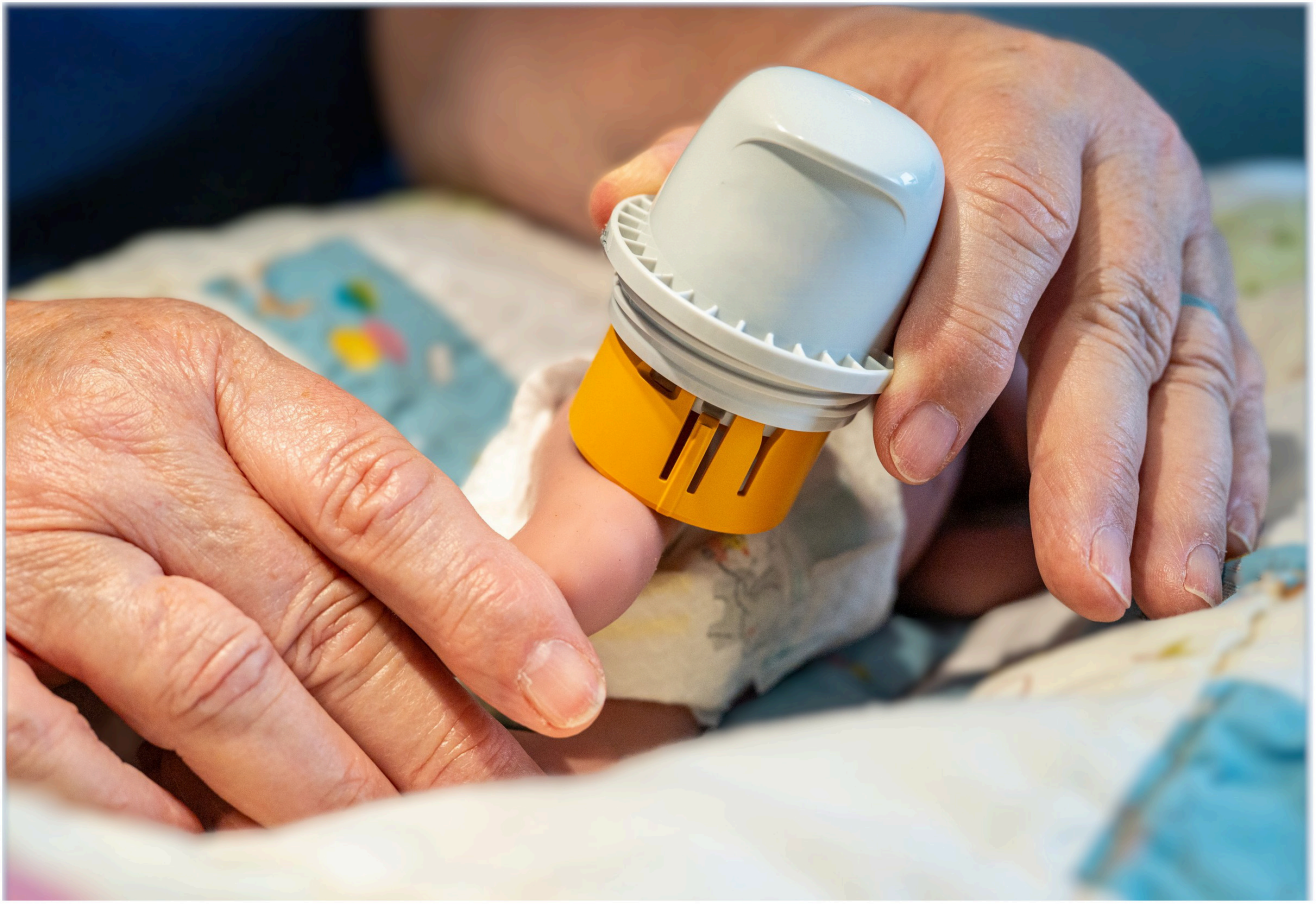
CGM insertion device in relation to a preterm infant. A standard commercial CGM device is held over the foot of a model infant, illustrating the scale challenge of a device not designed for use in the newborn.

The imminent concern is that manufacturers are moving toward integrated composite devices, in which sensor and transmitter are combined in a single spring-loaded applicator. These units are substantially larger, heavier, and more rigid than their predecessors, and their applicators are designed for adult or older-child anatomy. The combined device and inserter shown in Figure 2 illustrates the challenge. The size and force required for insertion mean these devices cannot safely be used in preterm infants. As manufacturers retire older devices in favor of integrated systems, the ability to use CGM even off label in neonates is under threat. This again highlights the need to develop purpose-built neonatal sensors.

What would a purpose-built neonatal CGM device need to look like? The requirements can be well defined and are technically achievable, though clinical validation and regulatory approval would of course present different challenges. Current sensors are designed to insert at 90° and to approximately 8 mm depth, reaching the subcutaneous fat of an adult or older child. Preterm infants have very little subcutaneous tissue, so a neonatal device would need to insert at a shallower angle and less depth. Fragile neonatal skin means limiting the adhesive footprint and using materials that do not damage preterm skin would be important. Sensors with factory calibration would also help to reduce the need for blood sampling. Validated accuracy at the hypoglycaemia threshold of 2.6 mmol/l would be essential for clinical utility, since reliable readings at this level would reduce the challenges of alarm fatigue.

## The neonatal CGM paradox

The clinical need is apparent, and the literature suggests the potential for CGM to be an important tool to better understand neonatal physiology, support short-term management and potentially impact on long-term health outcomes. However, without a device designed for use in the newborn, large-scale clinical trials of long-term efficacy are not feasible, and demonstration of clinical impact impossible to generate. Investment is needed to design and develop a bespoke neonatal device, but the hurdles for regulatory approval in a relatively small high-risk market are a barrier to investment. Without that evidence, there is no commercial case for developing a neonatal-specific device. Without industry investment, no licensing application is submitted. Without a device that can designed for the newborn it is challenging to undertake clinical studies to determine risks and benefits (20). This pattern is not unique to CGM: pediatric medical devices consistently lag a decade or more behind adult equivalents, with clinicians routinely making do with adapted adult technology (37, 38). With neonatal CGM, the clinical need is clear and the technical requirements of a purpose-built device are understood by neonatal teams.

Ironically the commercial trajectory is pushing devices into the healthy population with essentially no outcome evidence and no interpretive norms, with FDA providing over the counter clearance for non-diabetic use in children over the age of 2 years (39). In contrast, newborn infants at risk from hypoglycaemia and those in intensive care, are a population that may experience more severe and more clinically consequential glucose instability than the many others who have access to these devices. The inequality of access is not one of scientific challenge or clinical need, the technology demonstrably works in neonates, but without investment and regulatory approval these babies are being excluded from CGM technology which has brought about a step change in the management of people living with diabetes.

## The regulatory landscape

The challenges described are not unique to neonatal CGM. Pediatric medical devices consistently lag adult equivalents by a decade or more, with clinicians routinely forced to adapt adult devices in the absence of pediatric-specific alternatives (37, 38). This is not a problem that market forces alone will resolve. In December 2025, MedTech Europe, representing Abbott, Dexcom, Medtronic, Roche, and others, called for dedicated legislative pathways for breakthrough, orphan, and pediatric devices as part of MDR/IVDR revision (40).

Twenty years after the introduction of the Pediatric Regulation for medicines (41), the MDCG 2024-10 guidance on the clinical evaluation of orphan medical devices was published (42). This is the first dedicated EU framework acknowledging that MDR evidence standards can be disproportionately burdensome for small-population devices. It does explicitly permit off-label use data as legitimate clinical evidence for legacy orphan devices, but orphan status is strictly defined. The EMA subsequently opened a scientific advice pilot for orphan device designation and clinical evaluation strategy (43), and a dedicated expert panel for rare diseases and pediatrics was established under EU Decision 2025/1324 (44). The MHRA's Innovative Devices Access Pathway (IDAP) and proposed Early Access Service provide coordinated support for innovative devices addressing unmet NHS needs (45, 46). However, neither contains provisions specific to orphan or pediatric devices: there is no orphan device designation process, no adaptive clinical evidence standard. This gap sits in direct tension with the government's Life Sciences Sector Plan ambition to lead European life sciences by 2030, with rapid NHS adoption of innovative technologies as a central pillar (47).

The US FDA does provide specialized financial, regulatory, and testing pathways to accelerate the development of pediatric and rare disease medical devices. Key opportunities include the Pediatric Device Consortia Grants Program for funding and prototyping support, alongside the Breakthrough Devices Program to expedite regulatory reviews (59). Allowances like the Humanitarian Device Exemption lower clinical data requirements for conditions affecting fewer than 8,000 patients annually, while Early Feasibility Studies (EFS) permit small-scale clinical testing of early-stage designs. Furthermore, developers can utilize flexible clinical methodologies, such as Real-World Evidence and Adaptive Platform Trials, to reduce the time, cost, and safety risks associated with traditional pediatric clinical trials (48–50).

## Marginal market or an untapped opportunity

The argument that this is too small a market to justify investment deserves examination. The short and long-term costs of neonatal dysglycaemia are not small. There are a large number of term babies at risk from hypoglycaemia who require repeated blood sampling on busy postnatal wards and missed neonatal hypoglycaemia is costly to the family and to the NHS (51). Extreme preterm babies require care in NICU which is an expensive and limited resource. Any potential to impact on length of stay would have significant impact on health economics of purchasing CGM. Health economic analysis of the REACT trial found CGM to be cost-dominant: it was associated with lower costs and better outcomes across sensitivity analyses (28). However, long-term clinical outcome data requires large appropriately powered clinical studies.

Paradoxically the extremely high trans epidermal permeability of preterm skin at 23–26 weeks gestation may present an opportunity for non-invasive monitoring, an ongoing challenge in adult studies (52). Transdermal analyte extraction, near-infrared spectroscopy, and shallow microneedle sampling approaches that have failed in adult populations may prove viable in neonatal skin. Non-invasive, AI-supported multibiomarker monitoring may open new avenues for future clinical monitoring (53). The preterm infant's incomplete skin barrier may be precisely the environment where non-invasive glucose sensing which has never reliably worked in adults could first succeed.

In addition, although sensor accuracy is important less accurate measures of biochemical trends is typical in neonatal intensive care where many variables fluctuate rapidly and trends are valued to determine the need for more definitive testing. This is particularly important for preterm population where efforts are made to reduce definitive blood sampling to a minimum. The potential for CGM would be not for it to be used in isolation but as an adjunct to the better point accuracy obtained from blood glucose measurement, reducing the need for frequent blood sampling.

## Conclusion

Glucose instability in the newborn is common, often clinically silent, and carries serious consequences for neurodevelopment. There are similarities to the challenges faced by people with diabetes for whom CGM has dramatically impacted care. CGM has been shown to be feasible in the neonatal population, but further research and clinical studies are limited by devices not designed for newborns unique needs. These challenges facing neonatal CGM are not unique, they point to a broader issue affecting high-risk, rare-disease populations, where early commercial incentives fall short and support for derisking development is essential. The development of a neonatal CGM could set a precedent and demonstrate that needs-led device development for high-risk populations is achievable. It will however require sustained collaboration between clinical teams, research funders (NIHR), regulators, and industry.

## Data Availability

The data analyzed in this study is subject to the following licenses/restrictions: The data presented are derived from routine clinical care records and cannot be made publicly available due to patient confidentiality and data protection requirements. As anonymised retrospective clinical data, they are held under institutional governance and are not available for public deposition. Requests to access these datasets should be directed to Kathy Beardsall, kb274@cam.ac.uk.
